# The role of dispersal mode and habitat specialization for metacommunity structure of shallow beach invertebrates

**DOI:** 10.1371/journal.pone.0172160

**Published:** 2017-02-14

**Authors:** Iván F. Rodil, Paloma Lucena-Moya, Henri Jokinen, Victoria Ollus, Håkan Wennhage, Anna Villnäs, Alf Norkko

**Affiliations:** 1 Tvärminne Zoological Station, University of Helsinki, Hanko, Finland; 2 Baltic Sea Centre, Stockholm University, Stockholm, Sweden; 3 Havsfiskelaboratoriet, Swedish University of Agricultural Sciences, Uppsala, Sweden; University of Waikato, NEW ZEALAND

## Abstract

Metacommunity ecology recognizes the interplay between local and regional patterns in contributing to spatial variation in community structure. In aquatic systems, the relative importance of such patterns depends mainly on the potential connectivity of the specific system. Thus, connectivity is expected to increase in relation to the degree of water movement, and to depend on the specific traits of the study organism. We examined the role of environmental and spatial factors in structuring benthic communities from a highly connected shallow beach network using a metacommunity approach. Both factors contributed to a varying degree to the structure of the local communities suggesting that environmental filters and dispersal-related mechanisms played key roles in determining abundance patterns. We categorized benthic taxa according to their dispersal mode (passive *vs*. active) and habitat specialization (generalist *vs*. specialist) to understand the relative importance of environment and dispersal related processes for shallow beach metacommunities. Passive dispersers were predicted by a combination of environmental and spatial factors, whereas active dispersers were not spatially structured and responded only to local environmental factors. Generalists were predicted primarily by spatial factors, while specialists were only predicted by local environmental factors. The results suggest that the role of the spatial component in metacommunity organization is greater in open coastal waters, such as shallow beaches, compared to less-connected environmentally controlled aquatic systems. Our results also reveal a strong environmental role in structuring the benthic metacommunity of shallow beaches. Specifically, we highlight the sensitivity of shallow beach macrofauna to environmental factors related to eutrophication proxies.

## Introduction

Community ecology recognizes the joint influence of environmental and spatial factors in structuring communities [1,2]. The metacommunity, defined as a set of local communities connected by the dispersal of interacting species [1], is an emergent concept applied in community ecology when studying spatial patterns of biodiversity in aquatic systems *e*.*g*., [3–6]. The effects of environmental control and dispersal are crucial for structuring the community assemblages of organisms [1], and so far most of the metacommunity research has focused on the role of dispersal and the distribution and quality of habitat available to dispersers *e*.*g*., [5–8]. Thus, the relative importance of environmental and spatial factors has been related to the capacity of the species to display passive or more active dispersal mode [2]. Metacommunity studies in aquatic systems have classified those species able to disperse long distances and actively select preferred sites as strong dispersive species compared to a group of species in which dispersal is achieved through passive transport by, for instance, wind or animal vectors (*i*.*e*., weak dispersive species) [9–13]. In theory, weak dispersers are expected to show less environmental control and stronger spatial structuring, while strong dispersers exhibit greater environmental control and no significant spatial structuring (*e*.*g*., [4,6,11,13]). In open marine soft-sediment environments, many benthic invertebrates with pelagic larval stages are able to colonize new sites very quickly [2,14]. These organisms rely entirely on passive dispersal, mostly by water movement (*i*.*e*., waves, currents and tides), comprising a strong random component in their distributions [11,15–17]. However, many other benthic invertebrates without larval dispersal phase show strong independent mobility, thus, frequent small-scale dispersal is a common behavior for those adult invertebrates not permanently attached to the sediment and actively searching for preferred sites [16,17].

There is large variability in the dispersal mode of species in aquatic systems, which depends on the taxonomic and functional group considered [5,11,18–20]. Thus, recent surveys focusing on the metacommunity organization of different types of dispersers showed different results (see [12] for a review), highlighting the problems of generalization across aquatic systems. Therefore, it is important to apply different species categorizations using different traits of the individual species to obtain further insight of metacommunity dynamics. For instance, we can expect different conclusions if species are categorized according to their dispersal mode or habitat specialization [21]. Thus, some species show broad environmental tolerances (*i*.*e*., habitat generalists), while others have very specific habitat requirements (*i*.*e*., habitat specialists). These two categories have different population dynamics regardless of their dispersal modes [22]. The presence of appropriate habitats and the species ability to reach those habitats affect the degree of distribution of specialists and generalists [21,22]. Recently, metacommunity studies have started to consider the role of environmental and spatial factors in structuring aquatic invertebrates through habitat specialization [13,21]. Although the roles of dispersal and connectivity have been studied extensively in coastal marine ecosystems *e*.*g*., [23–26], here we combine for the first time both the species habitat specialization and dispersal mode to gain a better understanding of the underlying metacommunity mechanisms in an open marine system.

Most empirical metacommunity studies have focused on relatively small, controlled aquatic systems, such as lakes, ponds, or river-like systems [12,27]. However, the characteristics of the aquatic systems (*i*.*e*., ranging from single isolated lakes over stream networks to open marine coasts) differ markedly in their environmental heterogeneity, connectivity and spatial extent, suggesting different metacommunity organizations among major aquatic systems [12]. This large variability among aquatic systems will modify the way dispersal interacts with the prevalent environmental conditions. Theory suggests that environmental control prevails over spatial constraints in aquatic systems, and that the importance of dispersal limitation increases with the increasing spatial extent of the study area; consequently, distant sites likely support different ecological communities [1,12,28]. In marine systems, environmental control is also expected to have a significant role in structuring communities of organisms [2,12,14,29]. Among those environmental factors that may differ significantly between sites, eutrophication is a key stress factor causing biodiversity loss and homogenization of aquatic communities around the world [30,31], including the vulnerable shallow habitats of Baltic coastal areas *e*.*g*., [32,33]. Therefore, although the potential role of eutrophication in structuring metacommunities remains unknown, we can predict a strong sensitivity of shallow beach communities to eutrophication proxies. In addition, the role of the spatial component in the metacommunity organization of marine systems is expected to increase compared to freshwater systems (see [12]). Thus, in open coastal ecosystems, connectivity and dispersal rates among sites can be very high due to the action of waves and currents (*e*.*g*., [17,23,34]), especially compared to relatively isolated aquatic systems such as lakes without stream connections [11,12,18]. From a metacommunity perspective, this is relevant because connectivity and high dispersal rates tend to homogenize communities irrespective of their environmental conditions [1,12]. However, few studies in marine systems have assessed the issues of spatial scale under a metacommunity perspective, despite of having strong environmental gradients and being rather open to dispersal [15,16,29,35].

Metacommunity studies on the relative importance of environmental control versus spatial influence in structuring benthic invertebrates of coastal marine habitats are scarce [29,35], and such approaches have not yet been adopted for shallow marine soft-sediment areas. It is imperative to perform empirical studies in marine systems using similar approaches as in freshwater systems [12] to increase our understanding of metacommunity dynamics across major aquatic systems and to test predictions and the generality of existing findings. Our main aims were (1) to test the relative importance of environmental and spatial factors in structuring shallow beach benthic metacommunities and (2) to examine how different species categorizations (*i*.*e*., dispersal mode and habitat specialization) can affect the role of these two factors. We examine the hypothesis that dispersive species are more capable of tracking environmental variability compared to less dispersive species [4,5]. A strong link between specialists and environmental factors and between generalists and spatial factors has been demonstrated [21]. Therefore, we predict that environmental factors will be responsible for more of the variation in the abundance and distribution of beach specialists, while spatial factors will explain more of the variation in beach generalists.

## Methods

### Ethics statement

This study complied with all existing legislation governing animal welfare and field-based experiments. Animal ethics approval/permits were not sought as benthic invertebrate fauna manipulated/sampled in this study are exempt from the Animal Welfare Act 1999. For all sites, the landowner gave permission to conduct the study at the site. In addition, sites 1, 2, 3, 8, 9, 12, 13 and 19 were located in nature reserves and the permission for sampling was granted by the Centre for Economic Development, Transport and the Environment.

### Study area

**Fig 1:**
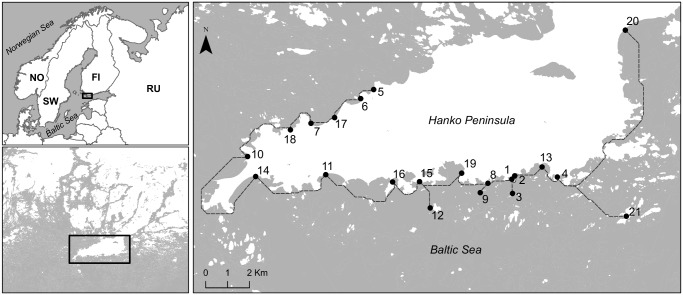
Map showing the location of the 21 beach sites around Hanko peninsula (Baltic Sea, Finland). The continuous dash-line is an example of coastline distances between sites simulating the hypothetical path of species dispersal across the seawater (following a GIS “cost-distance” raster, see Methods). “Countries, 2014—European Commission, Eurostat/GISCO.” Administrative boundaries: © EuroGeographics, © FAO (UN), © TurkStat Source: European Commission—Eurostat/GISCO. Contains data from National Land Survey of Finland Topographic map 1:100,000 downloaded 16.1.2017.

The study sites were located around Hanko Peninsula (59°N 23° E) at the entrance of the Gulf of Finland, in the northern Baltic Sea (Fig 1). The sites consisted of 21 shallow (depth max. 1 m) soft-sediment beaches characterized by a high exposure to wind and waves, and dominated by sandy substrates with a varying cover of algae and vegetation [36]. Shallow beaches around Hanko peninsula offer an excellent model system to test the consistency of metacommunity patterns across a diverse array of spatially connected local populations of macroinvertebrates. Beaches in this region provide well-defined open habitats, harbouring a number of invertebrates with different life histories and reproductive patterns that influence each species’ dispersal mode. This results in communities made of groups of invertebrate species with different traits [14, 21,31].

### Macroinvertebrate sampling

The field sampling took place during mid-August 2013 for a period of two weeks (sites sampled randomly) to match the seasonal peak in primary and secondary production. We collected five replicate sediment cores (Ø 5.6 cm, depth 15 cm) from each beach (0.3–0.8 m depth) to quantify soft-sediment benthic macrofauna. The sampling started from one end of the beach proceeding towards the other. The site average distances between consecutive samples ranged between 10–32 meters, approximately (18 ± 8 m; mean ± SD). We took sediment samples evenly over the whole site, and preserved them in 70% ethanol (stained with Rose Bengal) for later analysis. We sorted and identified macrofauna (after sieving, 0.2 mm) to the lowest taxonomic grouping practicable and compiled as total abundance. To minimize random effects by rare taxa, we selected taxa represented by at least three individuals in the total collection and present in at least two sites for statistical analyses. We provide a full taxa list and general descriptive statistics of the taxa used (Table 1). A site-specific summary of benthic infauna data and habitat characteristics can be found in [36].

**Table 1:** Descriptive statistics of the data set. AD: average density (individuals per core sample, area = 25 cm^2^) of each taxa across all beach sites, SE: standard error; B: niche breadth.

Species name	Counts^a^	AD	SE	Occurrence^b^	Dispersal mode^c^		Habitat specialization	
					Development	Type	B^d^	Type
*Bathyporeia pilosa*	151	1.47	0.41	8	Nonplanktonic	Active	11.71	Generalist
*Cerastoderma glaucum*	57	0.55	0.12	11	Planktonic	Passive	6.95	Generalist
Ceratopogonidae sp1	4	0.04	0.02	3	-	Passive^f^	2.67	Specialist
Chironomidae sp1	4303	41.78	6.25	17	-	Passive^f^	8.35	Generalist
*Crangon crangon*	1	0.41	0.16	1	Spawn/egg carrying	nc	1	nc
*Gammarus* sp.	42	0.1	0.05	9	Nonplanktonic	Active	4.25	Specialist
*Halicryptus spinulosus*	10	0.39	0.11	4	Nonplanktonic	Active	2	Specialist
*Hediste diversicolor*	40	0.07	0.02	12	Planktonic^e^	Active	7.41	Generalist
*Hydra* sp.	7	0.79	0.74	4	Planktonic	Passive	3.27	Specialist
*Hydrachna* sp1	81	1.31	0.27	1	-	nc	1	nc
*Hydrobia* sp.	135	0.04	0.02	12	Planktonic	Passive	7.22	Generalist
Hydrophilidae sp1	4	0.01	0.01	3	-	Active^f^	2.67	Specialist
*Idotea chelipes*	14	0.14	0.06	5	Nonplanktonic	Active	3.16	Specialist
*Limapontia capitata*	2	0.02	0.02	1	Planktonic	nc	2	nc
*Macoma balthica*	985	9.56	1.96	21	Planktonic	Passive	6.46	Generalist
*Marenzelleria* spp.	5	0.05	0.03	2	Planktonic^e^	nc	1.92	nc
*Mya arenaria*	2	0.02	0.01	2	Planktonic	Passive	2	Specialist
*Mytilus trossulus*	5	0.05	0.02	5	Planktonic	Passive	5	Specialist
*Neomysis integer*	11	0.11	0.07	3	Nonplanktonic	Active	1.46	Specialist
Odonata sp1	5	0.05	0.03	4	nc	Active	3.57	Specialist
Oligochaeta sp1	6460	62.72	11.04	21	Planktonic	Passive^f^	5.98	Generalist
Ostracoda sp1	3775	36.65	6.56	20	Planktonic	Passive	7.12	Generalist
*Piscicola geometra*	3	0.03	0.02	2	-	nc	1.8	nc
*Podura aquatica*	2	0.02	0.01	2	-	nc	2	nc
*Potamopyrgus antipodarum*	4	0.04	0.03	1	Nonplantktonic	nc	1	nc
*Prostoma obscurum*	46	0.45	0.09	16	Nonplanktonic	Active	9.29	Generalist
*Pygospio elegans*	1	0.01	0.01	1	Planktonic	nc	1	nc
*Stylaria lacustris*	6	0.06	0.03	2	Nonplanktonic	nc	1.8	nc
Trichoptera sp1	42	0.41	0.09	12	-	Active^f^	7.62	Generalist
Turbellaria sp.	103	1	0.39	7	Nonplanktonic	Active	3.42	Specialist
*Valvata* sp.	62	0.6	0.13	12	Nonplantktonic	Active	7.73	Generalist

^a^Number of records. Singletons and doubletons were not considered (nc) for further analysis.

^b^Number of sites where the species were present. For further analysis, we selected those species present in at least two sites.

^c^Information for dispersal mode obtained and combined from different sources (see Methods) and in-house knowledge. Not enough information, uncertainty, singletons and doubletons leads to nc.

^d^A detailed description on the niche breadth (B) metric can be found in the text (Methods: Habitat specialization). We arbitrarily selected 11 species with the greatest B (as generalists) and 11 species with the lowest B (as specialists) (see Methods).

^e^Lecitotrophic, species with short pelagic life (*i*.*e*., larvae in plankton during a few days) considered active dispersers (see Methods).

^f^Dispersal mode described for freshwater systems (see Methods) was used for categorization of specific invertebrate species.

### Dispersal mode: Passive vs. active

We used the typical dispersal mode categorization described for aquatic metacommunity studies (*e*.*g*., [5,11,37,38]), but adapted to a marine open system (*e*.*g*., [14, 31]). We broadly classified all the benthic invertebrates into two groups regarding different dispersal modes and larval development: (1) taxa with planktonic larval development and a long pelagic life, and (2) taxa with direct benthic, often brooding, larval development with a short or non-existent pelagic life. Then, we categorized taxa with planktonic larval development as passive dispersers (10 species), given that the maximal dispersal distance for planktonic larvae is mainly driven by water currents, and taxa with non-planktonic larval development as active organisms (12 species) with self-thrust movement and ability to select preferred sites. Although, there is evidence that dispersal distance of pelagic species is not purely determined by time in the pelagic phase, and that pelagic larvae have a reasonable degree of control over dispersal via behaviour, propagule duration is significantly correlated with dispersal distance [39]. Therefore, we expect active dispersers to show much shorter maximum dispersal distances (*i*.*e*., more dispersal-constrained) than passive dispersers, as the former will rely on their own energy to move. Active dispersers are more efficient at keeping their position than passive dispersers, as they are relatively more independent from vectors of dispersal (*e*.*g*., waves and currents) and can actively select for suitable habitats [16,21]. We used available information from the literature *e*.*g*., [14,21,31,40] and in-house knowledge to develop our classification. Additionally, aquatic insects were broadly identified to class or family, and then the dispersal mode described by [5] for freshwater systems was used to categorize them as active or passive (Table 1). This classification is a simple straightforward approach that allows comparing dispersal mode categorization among different aquatic systems.

### Habitat specialization: Generalist vs. specialist

We used Levins’ niche breadth (B) classification [41] to evaluate the importance of habitat specialization by calculating:

$$B_{j}=1/\overset{N}{\underset{i=1}{\sum }}P_{ij}^{2}$$

Where *B*_*j*_ is the niche breadth and *P*_*ij*_ is the proportion of the individuals of taxa *j* in a beach site *i*. We follow this method because it determines habitat specialization based on B as a function of uniformity of the distribution of taxa abundance among the beach sites for a specific community [26,41]. In theory, the calculated B is independent of the environmental variables within the study sites, or the spatial location of the different sites relative to each other in the landscape, necessary condition to avoid circularity issues [26]. Niche breadth for 22 taxa ranged from 1.00 to 11.71 (Table 1). Taxa with higher niche breadth values were those that used a wide range of sites (*i*.*e*., generalists), and taxa with lower niche breadth values were specialists. Since there is not a pre-defined threshold for identifying "high" or "low" B values [26], we arbitrarily selected eleven taxa with the lowest B as specialists (B = 1.46–5) and eleven taxa with the highest B as generalists (B = 5.98–11.71) for further analysis (Table 1). There was no significant relationship between average abundance and niche breadth (R^2^ = 0.054; *p* > 0.05), suggesting that taxa abundance was well mixed between specialists and generalists [26]. There was a different proportion of habitat preferences within passive dispersers (*i*.*e*., 60% generalists and 40% specialists) and active dispersers (*i*.*e*., 40% generalists and 60% specialists) (Table 1).

### Environmental variables

We measured different environmental variables to characterize each beach and to test relationships to macroinvertebrates, based on previous knowledge about their potential effect on the benthic habitats (see [42]). While the selected environmental variables are dynamic in both space and time, they have been demonstrated to (a) play important roles in “filtering” the distribution of invertebrate communities [11,16], and (b) serve as generic proxies for environmental quality (*e*.*g*., eutrophication) and habitat characteristics [36]. Moreover, the late summer period in August is the time-period following the annual recruitment for most benthic species and we would predict that the selected variables would have had time to influence the distribution and survival of juveniles.

We calculated a depth attenuated wind-wave exposure index for each beach from the GIS-based Isaeus-model [43], and we measured sampling depth (cm). Temperature (°C) and salinity (n = 10 replicates), and turbidity (FNU) (n = 5 replicates) were taken (mean values) close to the bottom at ca. 0.5 m depth along each beach.

A mean total cover (%) of vegetation and algae (including fast-growing filamentous algae) was calculated from 50 randomly taken 1 m^2^ quadrats (visually estimated) distributed evenly over each beach within the sampling depth range. For quantitative estimates of taxonomic groups and biomass (dry mass: 24 h in 60°C) a representative subsample of the vegetation and algae was obtained from 5 of the quadrats using a steel corer (Ø18.5 cm, sample area 270 cm^2^) [35]. Macrophytes and macroalgae biomasses per quadrat were related to the total cover by multiplying the biomasses with the proportion covered [36].

We analysed grain size and organic content from sediment samples (n = 5) collected regularly along each beach (0.3–0.8 m depth). We took grain size samples from the surface layer with a spoon (mean dry mass 87 ± 17 g) and we used hydrogen peroxide (H_2_O_2_, 6%) to dissolve organic material. The sediment samples were sieved into size class fractions (> 2 mm, >1 mm, > 0.5 mm, > 0.25 mm, > 0.125 mm, > 0.063 mm and < 0.063 mm, then dried (48 h, 60°C) and finally sediment mass was quantified. We calculated mean grain size (MGS) for each beach (log-phi Φ scale) using GRADISTAT [44]. We determined the organic content (samples taken from the top 1 cm of the sediment surface with a syringe, Ø 2.0 cm) as the loss on ignition (LOI: 3 h, 500°C) of dried sediment (%).

### Spatial variables

In complex marine systems, connectivity between communities is not a simple linear function of distance but, rather, is influenced by the geographic characteristics of the area [15,23]. Recent metacommunity studies have focused on estimating the effects of landscape resistance on the dispersal of organisms between sites *e*.*g*., [6,45]. We can obtain better ecological information using landscape resistance methods than typical straight-line distances. For instance, landscape resistance considers geographical barriers that affect the dispersal of species when calculating distances between communities [45]. The GIS term “cost-distance” has been successfully implemented as a proxy of landscape resistance in metacommunity studies since it takes into account a variety of factors that can affect the dispersal of organisms [6]. Resistance surfaces are typically calculated in a raster GIS-environment to understand how landscape characteristics influence connectivity.

We obtained the topographic data (as raster) of the study area (Hanko peninsula, Fig 1) from the National Land Survey of Finland (http://www.nls.fi). To estimate the distance between study sites, we created a variable (‘coastline’ distance) reflecting the geographical position of the beaches along the coastline (Fig 1). This approach offers a more realistic estimation of spatial connectivity than ‘overland’ distances since dispersal by seawater dominates in marine benthic environments. The geographical coordinates of the beach sites were imported into the ARCGIS 10.1 software (Esri, Redlands, CA, USA) and the determination of “cost-distances” was performed using the Path Distance tool in ArcMap modified by [6]. We reclassified the “cost-distance” raster to assign a “low-cost” value to the water area, and a “high-cost” value to the land area as an input to force the path across the seawater. The coastline matrix created was used as a distance matrix in the classical distance-based Moran’s eigenvector maps method (dbMEMs, formerly called Principal Coordinates of Neighbour Matrices, PCNM) to derive spatial variables (see [46,47]). We truncated the distance matrix to retain the distances between neighbouring beaches. Thus, the distances larger than a threshold value (*i*.*e*., the largest distance between two contiguous sites) were replaced by an arbitrarily large value equal to four times the threshold (see [46] for a detailed description). We computed a principal coordinate analysis of the truncated distance matrix and kept only the coordinates corresponding to positive eigenvalues. The resulting 15 principal coordinates (PCNMs) were used as explanatory variables in canonical redundancy analyses (RDA), computed for the Hellinger-transformed and detrended fauna data [48]. Significant PCNMs were identified by a forward selection procedure with unrestricted permutations for the community composition, and retained as spatial variables to be used in the variation partitioning analysis (DistLM). Calculations were performed using ‘vegan’ [49] and ‘packfor’ packages [47] in R software 3.2.2 [50].

### Data analysis: DistLM and variation partitioning

We used non-metric multidimensional scaling (nMDS) ordinations to visualize the variation in the environmental variability and taxa composition (Hellinger-transformed data) using the full set of samples (Euclidean resemblance matrices), and calculating distances from replicate site samples to centroids.

A distance-based linear model (DistLM, [51]) was performed to determine the proportion of variation explained by local environmental and regional spatial factors (selected as two different sets of predictor variables) on the benthic macrofauna community. DistLM performs variation partitioning in a similar way as in redundancy analysis or canonical correspondence analysis, generating *p* values by a permutation routine [51]. Three DistLMs, one for each type of taxa-response matrix (*i*.*e*., for the entire taxa matrix community, the dispersal capacity matrix and the habitat specialization matrix), were performed. Previously to DistLMs, we conducted a reduction in the number of the environmental variables to avoid undesired noise. The probability distribution of data for each variable and the multi-collinearity among variables was checked (Draftsman’s plot, [51]), and the whole set of environmental variables was reduced to eight main variables: exposure (log x+1 transformed), depth, temperature, salinity, turbidity, LOI, MGS, and total cover (algae + vegetation) (log x+1 transformed). Additionally, we related this reduced environmental matrix to each response matrix by means of the BEST procedure in PRIMER to select only those response-specific environmental variables. Finally, we built a site-characteristic matrix including values of the environmental variables and spatial variables to be used as two grouping factors in the DistLM procedure. DistLM was fitted using the forward selection procedure with sequential test (9999 permutations), and the best model was assessed using the Akaike information criterion (AIC). Small values of AIC indicating a better model [51].

DistLM is a multivariate extension of linear regression that measures the amount of variation (% of the total variation in the community matrix) that can be attributed to one or the other set of explanatory environmental ([E]) or spatial ([S]) variables. When testing for pure effects of spatial configuration ([S-E]), the set of environmental variables ([E]) was used as co-variable to remove its contribution to the explained variation. Similarly, when testing for a unique effect of environment ([E-S]), the set of spatial variables ([S]) was used as a co-variable. We also computed the total explained variation ([E+S]), the spatial structuring in the taxa data shared by the environmental variables ([E∩S]), and the unexplained variation (1-[E+S]) [2].

Since environmental variables can be considered the most significant drivers structuring invertebrate communities in aquatic systems [1,12,19], and due to the strong relationships between specific environmental variables and water eutrophication [32–34], a second round of DistLMs was performed to explore the specific contribution of each environmental descriptor on the composition of the macrofauna community. We applied distance-based redundancy analysis (dbRDA) to visualize the position of the beaches fitted to the significant environmental predictor variables affecting each type of taxa-response matrix. All multivariate analyses were performed with PRIMER 6^+^ PERMANOVA^®^ [51].

## Results

We found a total of 16,368 invertebrate individuals and 31 taxa across the twenty-one beach sites (Table 1). The average number of individuals per site (n = 5 cores) was 779 (SD = 646), and the average number of taxa per site was 6 (SD = 3). The nMDS representations of the environmental variables and community taxa (see supporting information, S1 Fig) of all the twenty-one beach sites sampled around Hanko peninsula (Fig 1) revealed a heterogeneous and diverse shallow soft-sediment beach network. We found a significant (*p* < 0.001), but weak relationship (6.1%) between community dissimilarity and environmental distance, and no significant relationship between community dissimilarity and geographic coastline distance (*p* > 0.05) (see supporting information, S2 Fig).

### Entire community partitioning

**Fig 2:**
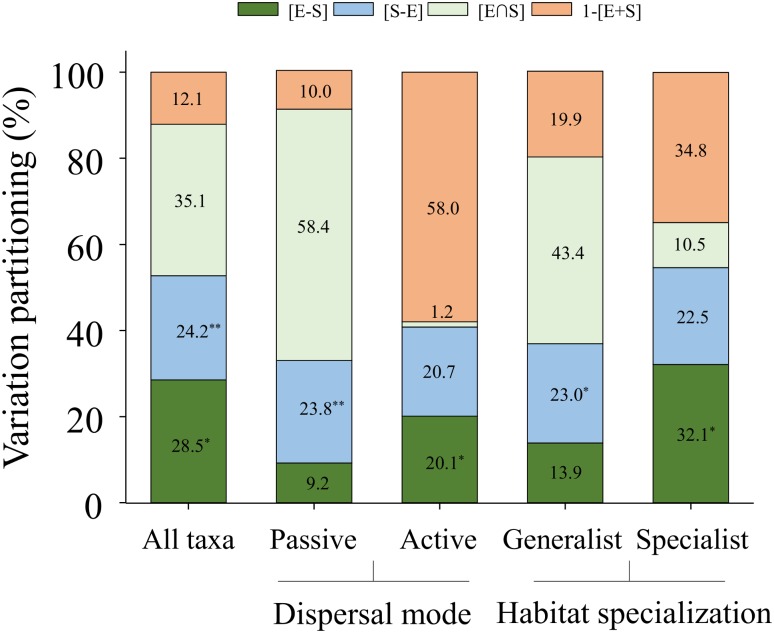
Variation partitioning (%) of the beach invertebrate taxa. Histogram showing all the invertebrate taxa plus four reduced data-matrices containing passive and active dispersers (dispersal mode) and generalist and specialist (habitat specialization) groups. All the models obtained using a forward selection procedure (DistLM sequential test, 9999 permutations). Components distinguished pure environmental variation [E-S], pure spatial variation [S-E], the variation component that is shared by both [E∩S], and the unexplained variation 1-[E+S]. *p < 0.05; **p < 0.01.

**Fig 3:**
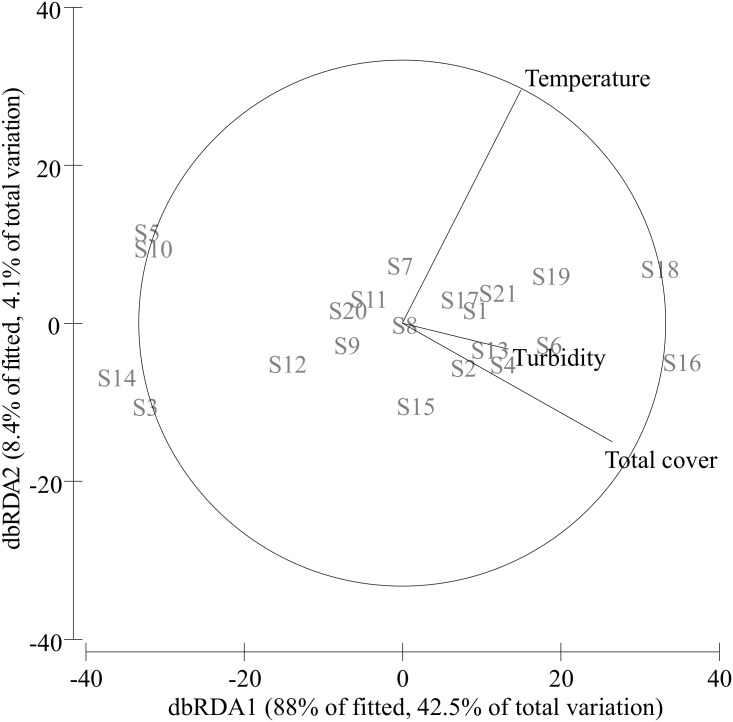
Distance-based redundancy analysis ordination. dbRDA visualizes the position of the 21 beach sites fitted to the main environmental variables (temperature, turbidity and total vegetation and algal cover) affecting the entire invertebrate community (see Table 3 for statistical results).

Overall, there was a significant effect of both environmental and spatial factors on the entire beach invertebrate community structure (Table 2). A combination of both pure environmental ([E-S]) and pure spatial ([S-E]) factors explained 52.7% of the variation in species abundance, with the pure spatial factor being a less influential contributor (24.2%; *p* = 0.006) than the environmental (28.5%; *p* = 0.016) factor (Table 2, Fig 2). According to the DistLM (the best model explained 48.3% cumulative variation) total cover, temperature, and turbidity were the main environmental variables explaining significantly the variation in the invertebrate community composition (Table 3, Fig 3).

**Table 2:** Variation partitioning explained (%) among environmental and spatial variables, and associated *p* values (variables at *p* ≤ 0.05 in bold) of the invertebrate data matrix for all the taxa, dispersal mode, and habitat specialization at the beach sites located around Hanko Peninsula (Baltic Sea, Finland). [E+S] = total explained variation by all variables in the model, [E] = variation explained by environmental variables, [S] = variation explained by spatial variables, [E-S] = pure environmental variation, [S-E] = pure spatial variation, [E∩S] = variation shared by environmental and spatial variables and 1-[E+S] = unexplained variation.

	All taxa		Dispersal mode				Habitat specialization			
			Passive		Active		Generalists		Specialists	
Variation	%	*p*	%	*p*	%	*p*	%	*p*	%	*p*
[E+S]	87.9	**0.006**	90	**0.003**	42	0.55	80.1	**0.023**	65.2	0.225
[E]	63.7	**0.005**	66.9	**< 0.001**	22	**0.02**	66.2	**< 0.001**	42.6	**0.002**
[S]	59.3	**< 0.001**	81.5	**< 0.001**	21.2	0.67	57.3	**0.001**	33.1	0.06
[E-S]	28.5	**0.016**	9.2	0.09	20.1	**0.04**	13.9	0.081	32.1	**0.018**
[S-E]	24.2	**0.006**	23.8	**0.005**	20.7	0.583	23	**0.023**	22.5	0.207
[E∩S]	35.1		58.4		1.2		43.4		10.5	
1-[E+S]	12.1		10		58		19.9		34.8	
AIC	127.5		122.6		170.4		126.7		171.7	

AIC = Akaike information criterion.

**Table 3:** Variation partitioning analysis (%) quantifying the sequential effects (stepwise selection, 9999 permutations) of the specific contribution of the environmental variables on the composition of the macrofauna community (significant results in bold at *p* ≤ 0.05). Total cover (algae + vegetation).

Invertebrate data matrix	Variable	SS	Pseudo-F	*p*	%	Cumulative
All invertebrate taxa	Total cover	3835.3	6.394	**0.001**	19.4	19.4
AIC: 137.9	Temperature	3237	3.728	**0.024**	16.4	35.8
	Turbidity	2464.8	3.162	**0.034**	12.5	48.3
Passive dispersers	Total cover	13118	21	**0.01**	52.5	52.5
AIC: 135.5	Turbidity	1454.1	2.72	0.07	5.8	58.3
Active dispersers	Exposure	5961.2	2.3	0.06	10.5	10.5
AIC:166.8	Organic matter	5979.4	2.4	**0.04**	10	21.2
Generalists	Total cover	5246.4	10.987	**< 0.001**	31	31
AIC: 132.3	Turbidity	3098	4.253	**0.025**	18.3	49.3
Specialists	Turbidity	10096	2.9538	**0.008**	13.5	13.5
AIC: 171.4	Temperature	6429.1	1.9778	0.069	8.6	22
	Depth	6086.9	1.9739	0.077^+^	8.11	30.1
	Organic matter	6617.7	2.3116	**0.041**	8.82	39

AIC = Akaike information criterion assessed model parsimony.

### Dispersal mode partitioning

**Fig 4:**
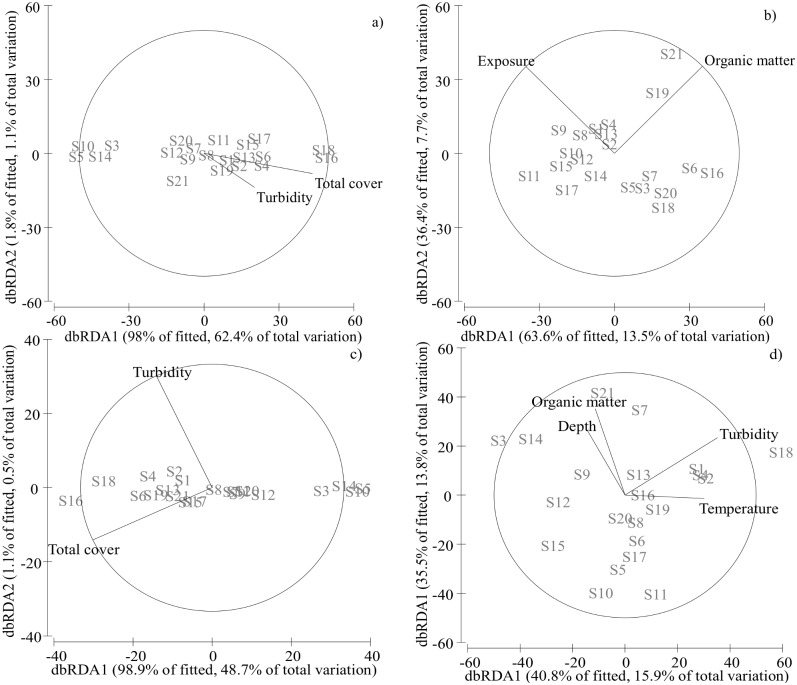
dbRDA (distance-based redundancy analysis) ordinations. (a) Main environmental variables (organic matter, turbidity and total vegetation and algal cover) affecting passive dispersers, (b) main environmental variables (organic matter and salinity) affecting active dispersers, (c) main environmental variables (turbidity and total cover) affecting generalist species, and (d) main environmental variables (temperature, turbidity, organic matter and depth) affecting specialist species (see Table 3 for statistical results).

Passive dispersers were significantly (*p* < 0.001) influenced by both environmental and spatial factors (Table 2). However, the variation in the abundance of passive dispersers was better explained by the spatial ([S] = 81.5 and [S-E] = 23.8%) than by the environmental components ([E] = 66.9 and [E-S] = 9.2%) (Table 2, Fig 2). The environmental explanatory variables (DistLM = 63.7%) influencing passive dispersers were total cover (*p* < 0.001), and turbidity (*p* = 0.07) (Table 3, Fig 4a). For active dispersers, only the environmental component ([E]) was significant, even after partialling out the spatial component ([E-S]) (Table 2). Thus, the variation in the abundance of active species was better explained by [E-S] (20.1%; p = 0.04) than by [S-E] (20.7%; *p* = 0.583) (Table 2, Fig 2). The best DistLM (21.2%) included exposure and organic matter as the environmental variables explaining significantly (*p* ≤ 0.06) the variation in the composition of active species (Table 3, Fig 4b).

### Habitat specialization partitioning

For generalists, a combination of both pure environmental ([E-S]) and pure spatial ([S-E]) components explained 36.9% of the variation, with [E-S] being a less influential contributor (13.9%; *p* = 0.08) than [S-E] (23.0%; *p* = 0.023) (Table 2, Fig 2). The environmental explanatory variables (49.3%) significantly influencing generalists were total cover (*p* < 0.001) and turbidity (*p* = 0.025) (Table 3, Fig 4c). A different picture emerged when we restricted the analysis to habitat specialists. Thus [E-S] accounted for 32.1% (*p* = 0.018) of the variation (Table 2, Fig 2). The main environmental explanatory variables (39%) influencing specialists were turbidity (*p* = 0.008), organic matter (*p* = 0.041), temperature (*p* = 0.069) and depth (*p* = 0.077) (Table 3, Fig 4d).

## Discussion

Metacommunity research in marine systems is still scarce, particularly about testing the relative importance of spatial and environmental patterns in governing variation in community structure [12]. Unlike lakes and streams, marine coasts are comparatively open systems with strong environmental gradients, where connectivity and dispersal rates are capable of moderating the role of environmental filtering *e*.*g*., [20,25,26,29]. Hence, marine systems are predicted to exhibit stronger spatial control compared to less-connected environmentally controlled aquatic systems (*e*.*g*., lakes and streams) [12]. Here, we used a shallow beach network system to test metacommunity predictions in such ubiquitous, dynamic and ecologically important coastal habitat.

### Benthic community structure in shallow beaches

We found both environmental and spatial factors to be important for explaining variations in benthic community structure of the shallow beaches. Similar to other aquatic systems (*e*.*g*., [3,4,6,29]), the prominent role of environmental factors suggests that a species-sorting perspective [1] may partly explain the metacommunity structure of the shallow beach network. Environmental variables related to eutrophication (*e*.*g*., total cover or water turbidity) explained a significant portion of the variation in the beach community structure. The occurrence of excessive submerged vegetation, including filamentous macroalgae, has increased during the last decades in the Baltic Sea due to eutrophication. Filamentous algae can act as a stress factor or as an alternative habitat for different species [32,52]. Turbidity (*i*.*e*., light deterioration in the water column), a proxy for eutrophication driven by anthropogenic inputs of nutrients, has increased during the last century affecting the photic depth and the structure of benthic communities, the distribution of aquatic vegetation, and the dynamics of the ecosystem [32,53]. Eutrophication has resulted in severe alterations of the community dynamics (e.g., biodiversity loss and biotic homogenization), affecting the functioning and the ecological value of coastal waters around the world [30–33,54,55]. For instance, nutrient enrichment induces enhanced primary production, leading to decreased photic depth, drifting mats of filamentous algae, and an elevated risk of low oxygen levels in coastal areas *e*.*g*., [32,52,56,57]. Temperature, which is reflective of relative water exchange in the shallow beach network, also explained a significant part of the variation in the beach community structure. Changes in water temperature can affect zoobenthos [55], and a warmer climate may increase hypoxia conditions amplifying the negative effects from eutrophication [58]. A better ecological understanding of marine benthic metacommunity dynamics can help improve predictions about the implications of eutrophication and biotic homogenization on coastal zones. This knowledge can help prioritize conservation actions to minimize biodiversity loss and improve management actions to cope with pervasive marine stressors.

Despite the important role of environmental factors, our results also suggest a significant role for spatial factors in explaining the metacommunity organization in open shallow beaches. A similar variance was explained by both pure spatial and environmental factors, while a large fraction of the community variation was explained jointly by both factors ([E∩S] = 35.1%) compared to a relatively small proportion of unexplained variance. Thus, environmental and spatial factors contributed similarly to the structure of the shallow beach communities suggesting that the interplay of environmental factors and dispersal-related mechanisms played a crucial role in determining the patterns observed [3,5,39]. Our study showed that spatial factors played an important role in structuring shallow beach communities compared to other aquatic systems, where they are generally less important (*e*.*g*., wetlands, [13,59]; lakes, [11]; streams, [5,60]), but see [4]. Although environmental and spatial factors regulated variation in the structure of the beach metacommunity, the relative strength of these factors varied depending on the categories of species traits used. For instance, we expected that different dispersal modes would have implications for the importance of environmental conditions affecting the community structure in connected systems [7,11,37].

### Dispersal mode

Active dispersers showed stronger environmental relationships compared to passive dispersers, suggesting that at the spatial extent of our beach network active dispersers were better able to track environmental variability than passive dispersers [4,5,11,37]. Although we classified active dispersers as less dispersive than passive species (*i*.*e*., dispersal-constrained), a significant correlation to environmental differences between soft-sediment beach sites is feasible considering the post-settlement capacity of many adults with direct benthic development to move around and select preferred sites [16,17]. In addition, many species without planktotrophic development can be passively transported or swim in the water column [26]. Specifically, organic matter, a well-known proxy for eutrophication in benthic environments, explained a significant portion of the variation in the structure of active dispersers. The low variation shared between spatial and environmental factors ([E∩S] = 1.2%), suggests a low correlation between spatial location and environmental characteristics; *i*.*e*., nearby beach communities do not necessarily share similar environmental conditions. It is important to note that some factors other than environmental or spatial factors contributed largely (1-[E+S] = 58%) to the variation partitioning in active species, potentially reflecting not-measured factors such as local biotic interactions [60].

Environmental and spatial interactions structured passive dispersers, supporting the idea of a joint influence of different types of metacommunity dynamics [2]. Interestingly, environmental factors were less influential contributors than the spatial factors for passive dispersers across our sites. Thus, environmental factors accounted for most of the variation in species with direct benthic development (*i*.*e*., active species), while spatial factors were significantly more important for those species with planktotrophic development (*i*.*e*., passive species). Previous studies support the existence of spatial signals on the structure of metacommunities of passive dispersers [59,61]. Planktotrophic invertebrates transported passively are frequently characterized as widespread dispersers [14,35]. This characterization may describe the potential for dispersal in some taxa, but it is not an exact generalization for dispersal rates [39, 61]. Thus, despite water movement, many benthic species are able to burrow or actively surface, regulating their post-larval transport along the seafloor [12,16,17,35]. We cannot discard that some of the spatial signal results from non-measured finer scale environmental variability [2], which could explain the shared variation observed for passive dispersers that would need further research. Our results suggest that high dispersal mechanisms are structuring the beach biotic community of shallow beaches [2,7,60]. Thus, water movement plays a key role on the community dynamics of marine systems, connecting distant areas and favouring dispersion of marine species at large scales [15,17,26,29,35]. High dispersal is likely to occur in sites that share similar environmental conditions and at spatial scales where connectivity is high [7,62], such as in our beach network. It seems feasible to think that physical distance is less important for species with high dispersal capacity because of their ability to reach suitable sites more often than organisms with low dispersal capacity [12]. However, we can find species in environmentally less-suitable sites due to intense water dispersal, concealing community-environmental relationships and resulting in a significant spatial signature in the partitioning analysis [5,7]. The variance not explained by either space or environment differed substantially between passive and active dispersers. Given that this corresponds to residual, site-to-site variability, it may also be a sign of strong spatial limitations at a scale shorter than the distance between neighbouring sites in a marine scenario where active dispersers are considered to show a shorter dispersal distance than passive dispersers.

Several studies that have used a similar approach for partitioning variation into different components have produced mixed results regarding which factors are important in determining metacommunity structure *e*.*g*., [4–6,11,62]. Dispersal can be highly variable in open coastal systems and strongly dependent on the functional traits of specific species [39]. Therefore, some factors other than dispersal can cause conflicting results, such as that different proportion of habitat specialists and generalists may occur among the dispersal mode groups [6,21,22].

### Habitat specialization

Our results strongly support the hypothesis that the spatial distance between beach sites influences variation of the abundance of generalists, and that local environmental factors influence variation of specialists. Thus, locally related mechanisms seemed central for specialists, for which environmental variables were especially important in structuring their local communities across beaches. Again, proxies for eutrophication (*i*.*e*., organic matter and turbidity) explained a significant portion of the variation in the community structure of beach specialists. For generalists, the spatial distance was the only significant factor explaining the largest contribution. Habitat generalists are adapted to utilize high environmental heterogeneity, since the environment is relatively uniform to generalists compared to specialists [21]. This hypothesis focuses on the idea that generalists are able to choose between different habitats that provide different resources and requirements. In coastal habitats, rates of dispersal can be especially high compared to other aquatic systems (*e*.*g*., lakes or streams), and many benthic invertebrate species are not permanently adhered to the substratum [23,34] and/or have a larval stage strongly influenced by waves and currents [17,20,23]. It seems appropriate to explain the observed patterns for beach generalists using metacommunity models that consider homogeneous environments emphasizing that stochastic patterns, such as dispersal mechanisms or ecological drift, with no environmental filtering, shape local communities [1]. Those few aquatic-ecology studies that have explored the interplay between environmental and spatial factors in explaining metacommunity structure through habitat specialization found similar results *e*.*g*., [13,21]. This is the first metacommunity study involving habitat specialization in the open-coastal context of a marine system, opening the possibility for further comparative studies on metacommunity organization across major aquatic systems [12].

## Conclusions

We argue that dispersal is likely to act as a major structuring force since beaches in our study area are situated relatively close to one another and linked through currents and waves. Unlike freshwater systems, environmental factors do not necessarily prevail in structuring marine systems, but also spatial factors (and therefore dispersal) play a main role in metacommunity dynamics. This supports the hypothesis that the role of the spatial component in aquatic systems is expected to increase following a theoretical increasing order of connectivity from isolated lakes over stream networks to coastal marine systems [12]. We provide a background to extend the metacommunity concept to marine habitats and to develop hypothesis about the determinants of species distributions across major aquatic systems. However, to understand fully the issues of dispersal and spatial scale applied to the modern metacommunity ecology approach more research on different marine systems, ranging from rocky and soft-sediment coasts to offshore and pelagic environments, are necessary.

The importance of environmental and spatial factors is not only dependent on dispersal mode, but also on the habitat use by specific species. We emphasize the complexity of benthic ecosystems, and stress the value of using different species categorizations in explaining variation in the abundance of macrofauna, as this approach allows a broader understanding of the underlying metacommunity structure. Our results showing that passive dispersers have some association with environmental factors suggest that different larval behavior can determine dispersal distance [39]. Thus, some of the passive species could also be specialists [13]. The proportion of specialists to generalists may restrict the results of those studies based either on the entire set of species or on the dispersal mode [21]. This supports the idea that different factors may govern contrasting groups of species within a metacommunity. This is ecologically important because specialists are more affected by habitat loss or homogenization than generalists, so different predictions for dispersal and habitat use under different metacommunity scenarios are necessary [21].

Different metacommunity models can explain those communities driven by purely spatial factors or without a clear role of the environment [1]. However, the real effect of spatial variation is difficult to interpret in empirical studies [7]. Broad-scale marine studies deal with multidirectional connectivity between sites, making the interpretation of dispersal limitation difficult [29]. It is key to understand the spatial structure of communities in a given coastline context to interpret the influence of multiple and interactive effects, including dispersal, species interaction, environmental variation and landscape heterogeneity in determining community structure [63]. On this matter, we successfully present a novel application of a landscape resistance modelling approach in ecological studies that consider realistic landscape variables that may yield more biological information on metacommunity structure not revealed by typical linear distance-models [6].

## Supporting information

S1 Fig Non-metric multidimensional scaling ordination of the 21 study beach sites. a) Environmental resemblance matrix (Euclidean distance calculated from replicate site samples to centroids) and b) Taxa resemblance matrix (Hellinger-transformed and Euclidean distance calculated from replicate site samples to centroids). S: stress.
(PDF)

S2 Fig Relationships between the community similarity matrix (Hellinger-transformed and Euclidean distance), environmental distance matrix (Euclidean distance) and distance matrix (nearest site to site distance), respectively. Each dot represents the relationship between 2 of the 21 beach sites and visualizes the relationship between two different distances (*i*.*e*. environmental distance, spatial distance or community similarity) between the considered beaches: a) Community similarity vs. Environmental distance (Estimate = -0.812, SE = 0.221, R^2^ = 6.1; *p* < 0.001), b) Community similarity vs Coastline distance (*p* > 0.05).
(PDF)
