# Supplementary material for: The role of dispersal mode and habitat specialization for metacommunity structure of shallow beach invertebrates
Source: PLoS One. 2017 Feb 14;12(2):e0172160. doi: 10.1371/journal.pone.0172160 (PMC5308789; doi:10.1371/journal.pone.0172160)

S1 Fig. Non-metric multidimensional scaling ordination of the 21 study beach sites. a) Environmental resemblance matrix (Euclidean distance calculated from replicate site samples to centroids) and b) Taxa resemblance matrix (Hellinger-transformed and Euclidean distance calculated from replicate site samples to centroids). S: stress.

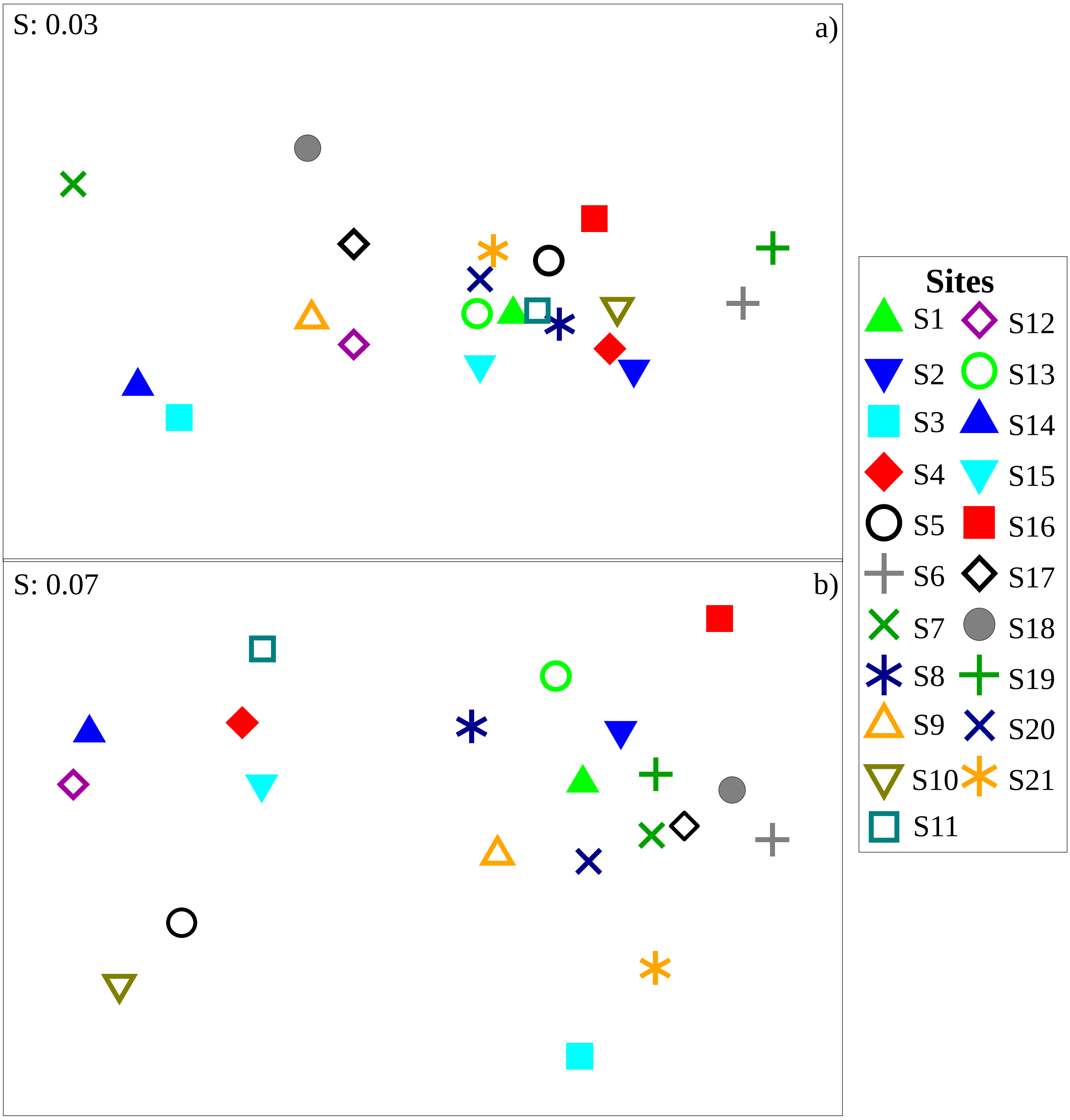

Supplement: S1 Fig — a) Environmental resemblance matrix (Euclidean distance calculated from replicate site samples to centroids) and b) Taxa resemblance matrix (Hellinger-transformed and Euclidean distance calculated from replicate site samples to centroids). S: stress. (PDF) [file pone.0172160.s001.pdf]
