# Supplementary material for: The role of dispersal mode and habitat specialization for metacommunity structure of shallow beach invertebrates
Source: PLoS One. 2017 Feb 14;12(2):e0172160. doi: 10.1371/journal.pone.0172160 (PMC5308789; doi:10.1371/journal.pone.0172160)

S2 Fig. Relationships between the community similarity matrix (Hellinger-transformed and Euclidean distance), environmental distance matrix (Euclidean distance) and distance matrix (nearest site to site distance), respectively. Each dot represents the relationship between 2 of the 21 beach sites and visualizes the relationship between two different distances (*i.e.*, environmental distance, spatial distance or community similarity) between the considered beaches: a) Community similarity vs. Environmental distance (Estimate = -0.812, SE = 0.221, R^2 = 6.1; p < 0.001), b) Community similarity vs Coastline distance (p > 0.05).

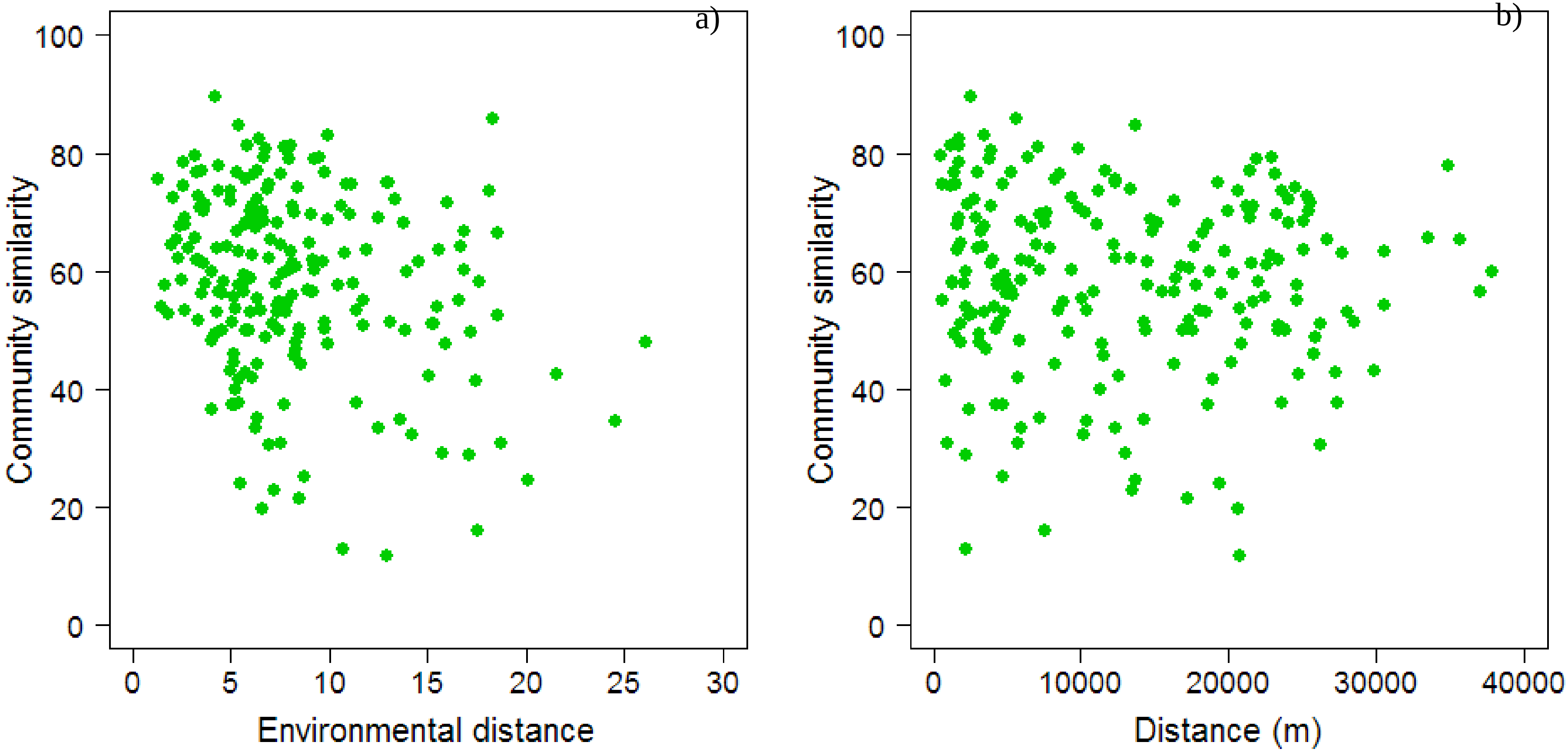

Supplement: S2 Fig — Each dot represents the relationship between 2 of the 21 beach sites and visualizes the relationship between two different distances (i.e. environmental distance, spatial distance or community similarity) between the considered beaches: a) Community similarity vs. Environmental distance (Estimate = -0.812, SE = 0.221, R2 = 6.1; p < 0.001), b) Community similarity vs Coastline distance (p > 0.05). (PDF) [file pone.0172160.s002.pdf]
